# Supplementary material for: Mitigation of the Adverse Effects of the El Niño (El Niño, La Niña) Southern Oscillation (ENSO) Phenomenon and the Most Important Diseases in Avocado cv. Hass Crops
Source: Plants (Basel). 2020 Jun 24;9(6):790. doi: 10.3390/plants9060790 (PMC7355666; doi:10.3390/plants9060790)
Supplement: Supplementary file 1 [file plants-09-00790-s001.pdf]

Supplementary files

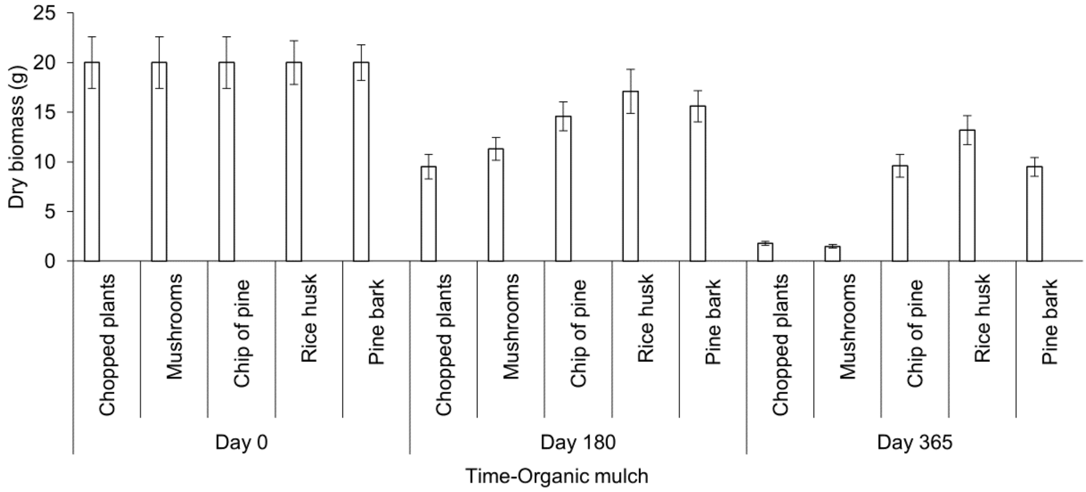

Figure S1. Decomposition of organic mulch through time. Error bars represent the confidence interval of the mean, validated by the Tukey mean separation test. No overlapping of the error bars indicates significant differences ($p > 0.05$).

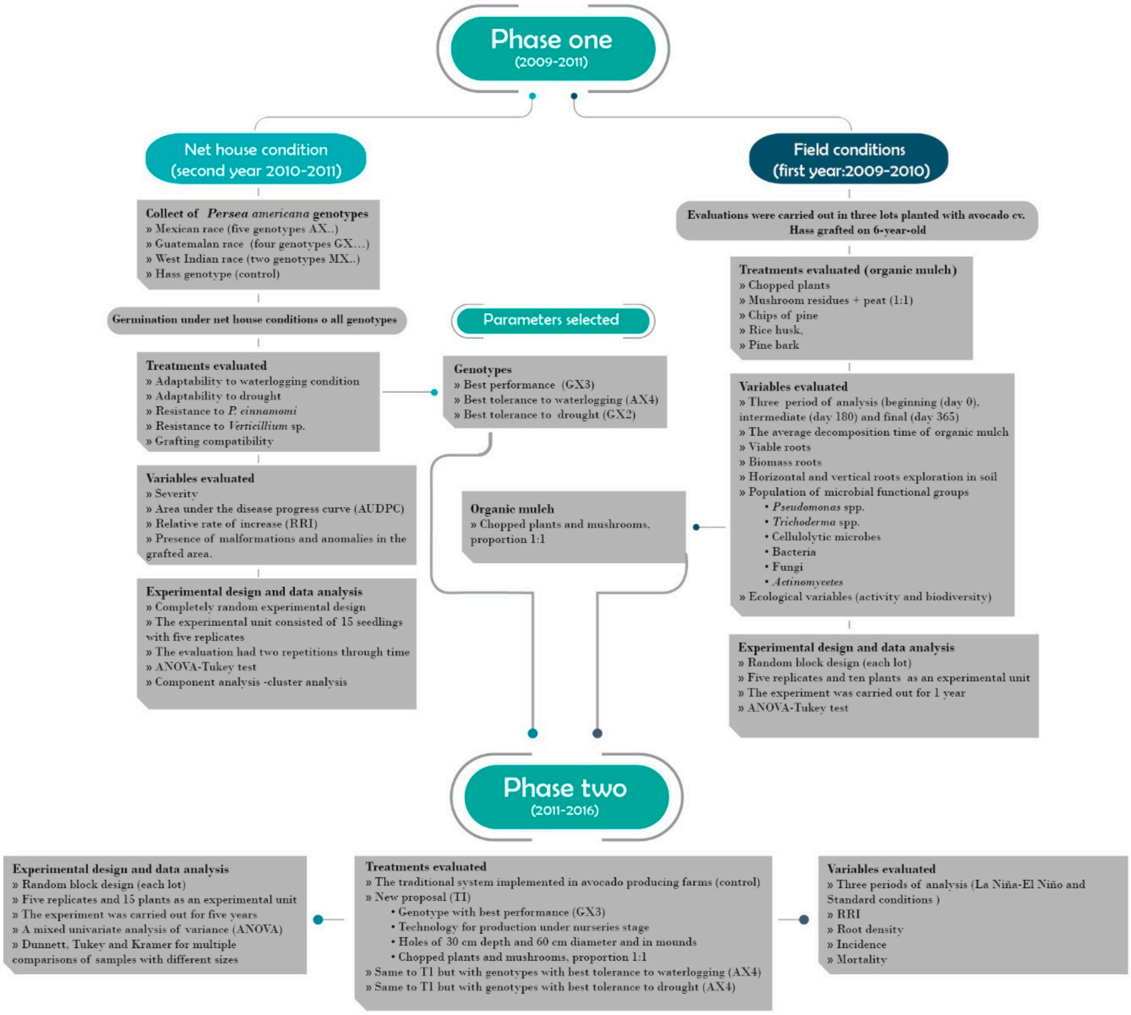

Figure S2. General scheme of experimental phases developed under net-house and field conditions.

Table S1. Edaphic variables in tested plots.

Soil	Sn[1]	Si[1]	Cla[1]	pH	OM[2]	Al[3]	Ca[3]	Mg[3]	K[3]	P[4]	S[3]	Cu[5]	Zn[5]	Mg[5]	B[5]
Don matias	52	28	22	5.2	12.3	0.6	2.55	1.1	0.45	21.3	5.1	0.8	2.3	1.9	0.3
El Retiro	60	22	18	5.3	14.8	0.8	3.5	1.3	0.55	24.4	5.6	0.9	1.9	2.5	0.5
La Ceja	58	23	19	5.5	13.1	1.6	1.9	0.9	0.35	15.9	5.9	0.9	2.4	2.1	0.35
Net-house	62	15	23	5.1	6.0	0.9	1.1	0.9	0.4	12.3	4.8	0.6	1.7	1.9	0.2

[1] Sand (Sn, %), silt (%), clay (Cla, %) (Bouyoucos). pH (water: soil, 1:2, *V:V*). [2] Organic matter content (OM, %) (Walkley and Black; aluminum). Aluminum (Al) (1M KCl). Calcium (Ca), magnesium (Mg), and potassium (K) (1M ammonium acetate). Phosphorus (P) (Bray II). Sulfur (S) (0.008 M calcium phosphate solution). Iron (Fe), manganese (Mn), copper (Cu), and zinc (Zn) (Olsen-EDTA buffer). Boron (B) (hot water). [3] Interchangeable bases (cmolc kg^{-1}). [4] mg kg^{-1}. [5] Available minor elements (mg kg^{-1}).
